# Does Partial Knee Replacement Allow Return to High-Demand Activities?

**DOI:** 10.7759/cureus.18439

**Published:** 2021-10-02

**Authors:** Hashim Al-Musawi, Mo Hassaballa, Jonathan Manara, Hywel Davies, Nick Howells, Damian Clark, Jonathan Eldridge, James R Robinson, Andrew Porteous, James Murray

**Affiliations:** 1 Trauma and Orthopaedics, University Hospitals of Bristol, Bristol, GBR; 2 Trauma and Orthopaedics, Southmead Hospital, Bristol, GBR; 3 Trauma and Orthopaedics, Bristol Royal Infirmary, Bristol, GBR; 4 Trauma and Orthopaedics, Avon Orthopaedic Centre, Bristol, GBR; 5 Orthopaedics, Avon Orthopaedic Centre, Bristol, GBR; 6 Orthopaedics and Traumatology, Avon Orthopaedic Centre, Bristol, GBR; 7 Translational Health, University of Bristol, Bristol, GBR; 8 Trauma and Orthopaedics, North Bristol NHS Trust, Bristol, GBR

**Keywords:** patient reported outcome measures, high demand activities, partial knee replacement, function improvement, oxford knee score

## Abstract

Purpose

The purpose of this study was to assess postoperative partial knee replacement (PKR) functional improvement using the postoperative Oxford Knee Score for Activity and Participation Questionnaire (OKS-APQ). PKR includes medial, lateral, and patellofemoral knee arthroplasty.

Methods

A search of a National Health Service hospital database was made to identify eligible candidates for a survey of Patient-Reported Outcome Measure (PROM). Database records were collected for patients who had medial, lateral, and patellofemoral knee arthroplasty. The first author, an orthopaedic surgery resident, retrospectively reviewed the data and selected 318 patient records for inclusion in a questionnaire survey. The inclusion criteria were: patients who had PKR within three years from the time of the study and patients who don’t have medical problems that may affect their mobility; for example, balance problems. The survey used the postoperative Oxford Knee Score for Activity and Participation Questionnaire (OKS-APQ), Tegner Activity Score (TAS), and four questions were added to the present study, namely, three free-text questions and one visual analogue score (VAS). The survey was sent by post seeking the patients' responses.

Results

Two-hundred five responded to the survey out of 318; a 64% response rate. The ceiling and floor effects were determined from patients’ answers. Survey questions included: What is the most demanding activity you routinely do every month on your new knee? The patients’ answers were divided into four groups. First, 29% were limited to low functional demand activities, for example, light walking for less than a mile. Second, 43% were involved in domestic work and sports activities, for example, golf, skittles, bowling, squatting, swimming, and gardening. Third, 21% had progressed to higher demand activities, for instance, dancing, racquet sports, cycling, and yoga. Fourth, 7% were performing higher demand activities involving impacts, for example, skiing, heavy gym workout, and marathon running.

Conclusion

The postoperative questionnaire demonstrated activities ranging from high-impact activities, for example, skiing, and from higher demand activities, for example, dancing to low function activities, for example, light walking.

## Introduction

This study employs the joint-specific postoperative activity and participation questionnaire (APQ) supplement to the Oxford Knee Score (OKS). It is commonly used to assess the outcome of total knee replacement Patient-Reported Outcome Measure (PROM) [1]. The purpose of this study was to assess postoperative PKR function improvement using OKS-APQ.

Recent studies reported results for PKR, unilateral knee replacement (UKR) [1-10], contrasts between mobile-bearing versus fixed-bearing, and cemented versus cementless implants. It has been suggested that UKR improves range of motion and functional recovery as well [4-19]. The term PKR is used to describe the replacement of the medial compartment, lateral compartment, or patellofemoral joint (PFJ). It is generally agreed that PKR offers good function enhancement [2-12]. Furthermore, PKR is a well-established treatment option for end-stage unicompartmental (medial, lateral, or patellofemoral) osteoarthritis of the knee, after the failure of conservative measures. PKR shows good function enhancement compared with total knee arthroplasty (TKA), and recent evidence recommends it as the first line for selected patients. The National Joint Registry shows the number of PKR performed varies significantly amongst surgeons [15-16]. It has been shown that high-volume PKR surgeons and units have better outcome data and that surgeons should perform more than a minimum number of partial knees a year to show good implant longevity [8-14]. Some studies have quoted an improved range of movement and PROM as well as returning to their preferred sporting activity [3-13].

There has been an increasing interest in maximum function with more modern PROM scores trying to address ceiling and floor effects [1-17]. However, there is little data available as to what specific level of activities that patients are able to return to following PKR [3-18]. Many studies focus on PROM data and survivorship as outcome measures [11-14]. Our aim is to look at the highest functional activity possible for patients.

## Materials and methods

A search of an NHS hospital database was made to identify eligible candidates to participate in PROM. The database records related to medial, lateral, and patellofemoral knee arthroplasty. So, the first author (HA), a trainee orthopaedic surgeon, retrospectively reviewed the data and selected 318 patient records for inclusion in a questionnaire-based survey. The survey uses the postoperative Oxford Knee Score supplement for Activity and Participation Questionnaire (OKS-APQ), Tegner Activity Score (TAS); four questions were added for the present study, namely, three free-text questions and one visual analogue score (VAS). The survey was mailed, seeking patients' responses. The questionnaire has been validated by the Oxford Knee Group, Oxford University, United Kingdom. Data collected from the validated questionnaire have been extracted for analysis.

For the recommended clinical indications, 205 PKRs were performed by seven consultant surgeons in the time span from January 2016-December 2018. There were 97 males (47%) and 108 females (53%). Database records for candidates for the survey were retrospectively reviewed. This includes medial, lateral and patellofemoral knee replacements. The Knee Research database was reviewed to identify all eligible patients. Postal questionnaires were sent to all eligible patients asking them to complete the following: the Oxford Knee Score Activity and Participation Questionnaire (OKS-APQ) (14 questions), visual analogue score, and Tegner Activity Score (TAS).

Additionally, there were three free-text questions. The first question asked what was the most demanding activity the patient had ever performed on the operated knee. The second question addressed the most demanding activity routinely performed on the operated knee every month. The final free-text question asked patients to list activities that they are unable to perform as a consequence of the surgery on the knee.

The senior authors subsequently categorised patients into four groups according to knee function development; varying from low to high demand. Non-responders were contacted after eight weeks, results were collected via telephone, and VAS was calculated by asking to score from 1-10 how satisfied they were with their knee. Demographic data were collected, as well as the compartment replaced and a prosthesis used. 

All seven surgeons in our unit who perform PKR were included and all procedures were performed by them or under their direct supervision. We limited our time-collection period so that all patients were between one- and three years of follow-up. This allowed full recovery from the procedure but reduced the potential for age-related decline in function and the risk of acquiring additional musculoskeletal comorbidity. Other exclusion criteria were patients with medical co-morbidities that prevented activity level beyond walking, patients with cognitive impairment that prevented them from being able to complete the questionnaire and any bicompartmental procedures, e.g., medial and PFJ.

From cross-referencing consultant-level reports of the individual surgeons, this represented approximately 45% of their total partial knee surgeries. In the PKR replacement volume for the time period 2016-2019, patients were operated on by seven specialist knee surgeons, meaning all surgeons in the present work; a mean of 29 patients per surgeon (range 20-105).

## Results

**Figure 1:**
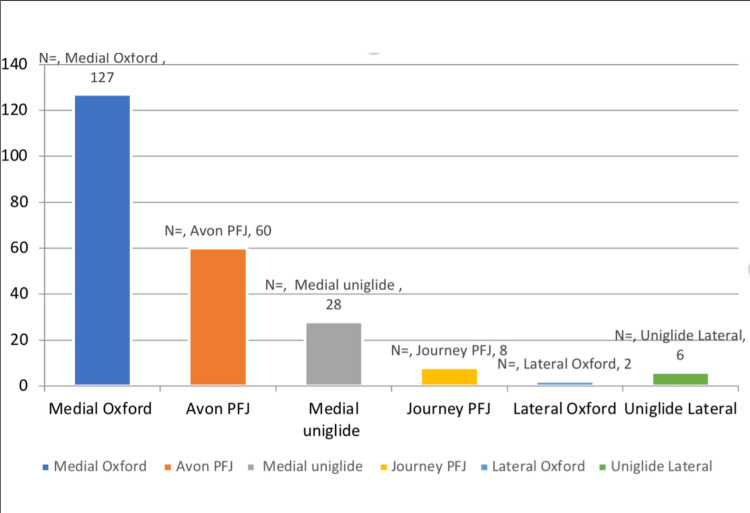
Responders according to the prosthesis

**Figure 2:**
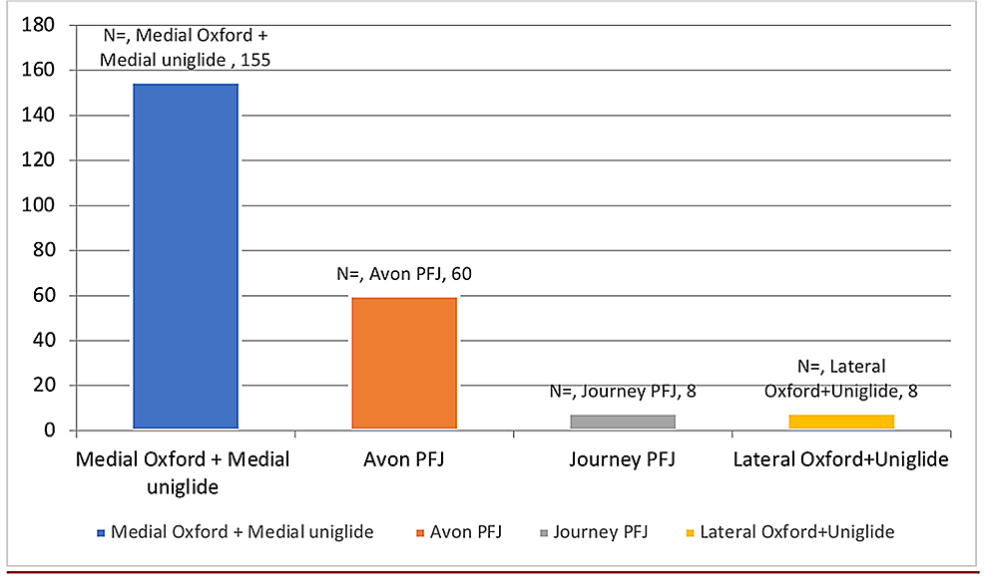
Number of patients with each prosthesis

Figure 1 shows the responders according to the type of prosthesis while Figure 2 shows the number of patients using each prosthesis.

Two-hundred five responded to the survey out of 318; a 64% response rate. The postoperative ceiling and floor effects were determined from patients’ answers. The preoperative evaluation was not included in this study. Survey questions included: What is the most demanding activity you routinely do every month on your new knee? The patients’ answers were divided into four groups. First, 29% were limited to low functional demand activities, for example, light walking for less than a mile. Second, 43% had involvement in domestic work and sports activities, for example, golf, skittles, bowling, squatting, swimming, and gardening. Third, 21% progressed to higher demand activities, for instance, dancing, racquet sports, cycling, and yoga. Fourth, 7% were performing higher demand activities involving impact, for example, skiing, heavy gym workout, and marathon running.

The time from surgery to completion of the questionnaire was a median of 2.6 years with a range of one to three years. One patient was subsequently excluded from the study, as their functional deficit was due to other medical conditions.

With regard to the three free-text questions, once the whole cohort of responses had been returned, we categorised them into four separate groups. The first group was of patients (low functional demand) in whom activities were limited to light walking of less than a mile. The second group expressed more involvement in domestic work and activities such as golf, skittles, bowling, squatting, swimming, and gardening. The third group had progressed to higher demand activities such as dancing, racquet sports, cycling, and yoga. The fourth group of patients were able to perform the highest demand activities involving impacts such as skiing, heavy gym workouts, and marathon running. The average Oxford APQ score was 22, median 27, range 6-32. The average VAS satisfaction was 8, median 9, and range 1-10. The TAS was completed fully in 59% of cases (all other questionnaires had a 100% response rate). The average and median TAS was 3. Kneeling was the main functional deficit (48%). The free-text questions were complete in all cases and demonstrated a range of activities between patients. The senior authors decided what constituted a group. Age and sex influence have not been evaluated in this survey.

## Discussion

The most important finding of our study is the excellent functional outcomes achieved using PKR. Our study shows that post-PKR, patients can achieve high-demand activities. Patients could, postoperatively, participate in a diverse range of high functional demand activities. Post-PKR Group Four, 7% of the cohort, performed higher demand activities involving impact, for example, skiing, heavy gym workouts, and marathon running. On the other hand, 21% of the cohort, Group Three, performed higher demand activities, for instance, dancing, racquet sports, cycling, and yoga. Group Two, 43% of the cohort, had involvement in domestic work and sports activities, for example, golf, skittles, bowling, squatting, swimming, and gardening. Group One, 29% of the cohort, were limited to low functional demand activities, for example, light walking for less than a mile.

There appears to be a difference between the compartments replaced, with medial PKRs achieving the highest functional scores PFJs and laterals. Our results agree with PKR outcomes in the literature. Our cohort had 43% in Group Two, who performed at least light sports, indicating that postoperative function is higher following PKR. Yapp et al. described patients’ ability to perform outside activities following surgery and demonstrated persistently low levels of fulfilment; two-thirds of patients were unfulfilled by their walking ability at one year. Furthermore, patients who had PKR were satisfied with a visual analogue score mean of 8, showing higher satisfaction. Wilding et al. showed the mean satisfaction score as determined by a visual analogue scale of 7.6 [16]. Hauer et al. suggest that unilateral knee arthroplasty UKR is associated with higher activity level, higher quality of life, and greater range of movement [6]. A recent systematic review showed PKR as a viable option for the treatment of isolated unicompartmental osteoarthritis [1]. Other studies have looked at functions after PKR. Fisher et al. looked into the sporting and physical activities of 76 patients who had undergone Oxford medial unicompartmental knee arthroplasty. They reported excellent outcomes, with 93% of patients successfully returning to their regular sporting or physical activities following surgery [5]. In their study, “regular sporting and physical activity” was defined as taking part in any given sport at least twice a month. Hooper et al. compared the sporting activity of 76 and 34 PKR patients postoperatively and found 85.3% of PKR patients participated in low-impact sports (bowling, golf, and swimming) after surgery [17]. This is similar to the 91% of patients that are able to achieve low demand, light sports, and moderate sports activities in our cohort. Walker et al. looked into surveying the activity level and the health-related quality of 45 patients following PKR at a mean follow-up of three years; their results showed that two-thirds reached a high level of activity counting the number of sports activities. Witjes S et al.'s systematic review and meta-analysis showed 75-100% of PKR patients return to sport, and physical activity was higher after PKR than after TKR, but a trend towards lower-impact activity was shown after PKR and TKR [17]. Studies on high-impact sport (tennis/squash, skiing, running) are rare in literature [11], but our findings suggest it is an achievable goal in over a quarter of patients undergoing PKR, especially medial compartment replacement. We also found that patients' Oxford APQ score does not necessarily correspond with the satisfaction of the patient with 82 achieving a TAS of 3 but having a VAS of 8 or above. This is a reminder that low-demand patients can also be appropriate and satisfied with a PKR. In our study, patients with lateral knee replacement do not do as well as with medial knee arthroplasty, achieving high-level activity, six patients fell below Group Two and one patient under Group Three, which is a small number of patients compared to 47 medial knee replacements. There are many reasons why this may be the case. Smith et al. stated that isolated unicompartmental knee arthritis is less common laterally than medially and lateral unicompartmental knee arthroplasty (UKA) constitutes only 1% of all knee arthroplasties performed [16] and so could be a factor to do with experience in performing the procedure or the disease process itself.

The limitations to our study were that it involved a single centre and different surgeons using different prostheses and the included patients were in a narrow post-operative window. The influence of age and sex have not been evaluated because they were not part of the research design.

The main addition to the literature from the present study is that patients with PKR are capable of performing high-function activities; we can inform patients that 28% can expect to achieve a high level of function. Return to high functional demand sports activity was the most significant finding of our study. The highest was in Oxford medial knee replacement, at 8%. PKR was at a much lower percentage.

## Conclusions

The postoperative Oxford Knee Score Activity and Participation Questionnaire (OKS-APQ) demonstrated a range of Patient-Reported Outcome Measures (PROM) in the present study, ranging from performing higher demand activities involving impact, higher demand activities without impact, domestic work and sports activities, to limited to low functional demand activities.

The present study confirms results from the literature relating to partial knee replacement PROM. It also verifies that patients do well functionally after PKA. Furthermore, our study showed that partial knee replacement enabled patients to achieve high functional demand activities.
